# Applications, challenges and prospects of mesenchymal stem cell exosomes in regenerative medicine

**DOI:** 10.1186/s13287-021-02596-z

**Published:** 2021-09-28

**Authors:** Aysa Rezabakhsh, Emel Sokullu, Reza Rahbarghazi

**Affiliations:** 1 Cardiovascular Research Center, Tabriz University of Medical Sciences, Tabriz, Iran; 2 Koç University Research Center for Translational Medicine (KUTTAM), Rumeli Feneri, 34450 Sariyer, Istanbul, Turkey; 3 Stem Cell Research Center, Tabriz University of Medical Sciences, Tabriz, Iran; 4 Department of Applied Cell Sciences, Faculty of Advanced Medical Sciences, Tabriz University of Medical Sciences, Daneshgah St., Tabriz, 5166653431 Iran

**Keywords:** Exosomes, Whole-cell therapy, Disadvantages, Challenging, Regenerative medicine

## Abstract

Recent advances in the identification and application of different stem cell types have offered alternative therapeutic approaches for clinicians. The lack of successful engraftment, migration into the injured site, loss of functionality and viability, ethical issues, shortage of donated allogeneic stem cells and the possibility of transmission of infectious are the main challenges associated with direct cell transplantation. The discovery and research on exosomes have led to the rise of hopes for the alleviation of different pathologies in regenerative medicine. Exo are nano-sized extracellular vesicles (40–150 nm) and released by each type. These nanoparticles participate in cell-to-cell communication in a paracrine manner. It is thought that the application of Exo can circumvent several drawbacks related to whole-cell therapies. Because of their appropriate size and stability, Exo are touted as therapeutic bullets transferring signaling factors into the acceptor cells in a paracrine manner. Despite these advantages, technologies associated with Exo isolation and purification are challenging because of heterogeneity in exosomal size and cargo. The lack of standard GMP-grade protocols is the main hurdle that limits the extensive application of Exo in the clinical setting. Here, the authors aimed to inspire a logical and realistic vision about problems associated with Exo application in regenerative medicine.

## Background

The discovery of stem cells has paved a way to accelerate tissue regeneration via replacing injured cells in paracrine and juxtacrine manners. Among several stem cell types, MSCs exhibit significant trans-differentiation properties into several lineages after transplantation into the target sites [1]. Due to the ease of extraction, presence in most tissues and differentiation into several lineages, MSCs are top-used cells in regenerative medicine compared to the other stem cell types [2]. These cells can be isolated from bone marrow, umbilical cord blood and adipose tissue. To use MSCs in the clinical setting, it is mandatory to expand freshly isolated MSCs in large scales in vitro. Unfortunately, these features can lead to genetic and morphological alterations likely after several passages [3]. Indeed, obtaining cells with similar and typical characteristics is not completely controllable. The viability of transplant cells, migration into the injured sites and integration with host cells are the main hurdles affecting the efficiency of cell-based therapies [4]. It is noteworthy to mention that a large number of exogenously administered MSCs are eliminated irrespective of immune system reaction due to mechanical stress and lack of supporting niche [4]. As a correlate, these drawbacks have forced the researchers to focus on other aspects of stem cell-based therapies.

In addition to the differentiation capacity, stem cells especially MSCs can release several signaling molecules inside nano-sized vesicles namely Exo, ranging from 40 to 150 nm, to remotely regulate the behavior of acceptor cells Exo are can be found in biofluids containing diverse signaling molecules such as miRNA, mRNA, lipids, DNA and proteins [5]. Several experiments have shown that a wide range of MSCs isolated from different tissues display variable Exo secretion capacity [6]. Molecular investigations showed that over 50% of cargo is common between Exo isolated from various types of MSCs [6]. As mentioned previously, a large amount of cargo in MSC Exo correlates with the regulation of cell growth and antioxidant activity [7]. The procedure of Exo biogenesis is intricate and encompasses several consecutive steps inside the cells. In brief, the phenomenon consists of the generation of the endosomal compartment, namely MVBs, from the trans-Golgi apparatus and the invagination of intraluminal vesicles into the lumen of multivesicular bodies [8]. Exo possess high stability during preparation steps and lyophilization. Recent works exhibit immune tolerability even with repeat administration. Due to the cell-free nature, Exo exhibited a better safety profile in which side effects and toxicity after Exo administration is approximately unlikely [9]. Of note, both xenogeneic and allogenic Exo can be administrated without provoking immune system reactivity [10]. Compared to MSCs, systemic injection of Exo leads to proficient delivery to the injured site without affecting structural integrity [11]. Compared to their parent MSCs, the levels of systemically injected Exo from natural barriers are higher. The existence of distinct ligands on the exosomal membrane and ligand-mediated endocytosis makes these particles eligible for delivery purposes [12]. Commensurate with these descriptions, Exo have numerous superiorities over MSCs in the regeneration of injured sites. Here, we aimed to highlight some issues and problems related to Exo isolation, purification and application in in vivo conditions.

## Challenges related to Exo application

**Fig. 1:**
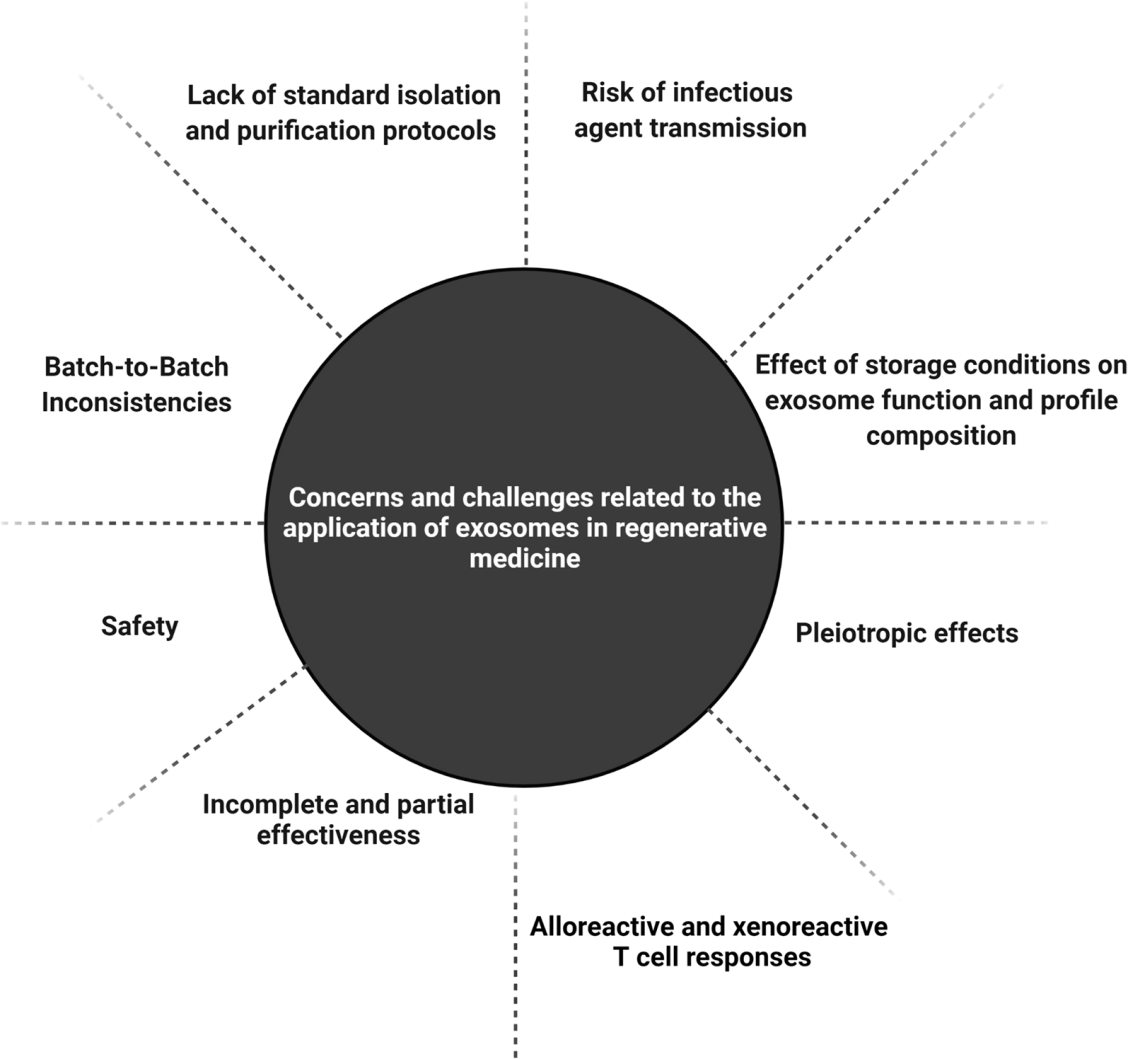
Several challenges are associated with the application of Exo in the clinical setting

While one might hypothesize that Exo are touted as a consolidated therapeutic approach for many, if not most, diseases, the reality is that the application of Exo is at the primitive steps of development for clinical purposes despite putative advantages (Fig. 1 and Table 1). Up to now, different approaches such as precipitation, ultracentrifugation, ultrafiltration, flushing separation, microfluidic isolation, antibody affinity capture and mass spectrometry have been developed for Exo isolation from biological fluids [6] (Table 2). Noteworthy, these techniques are, indeed, laborious, time-consuming and expensive without established protocols. For example, the existence of natural components such as chylomicrons and lipoproteins can affect the isolation of Exo from biofluids [13]. The lack of typical surface markers and the existence of other extracellular vesicle types such as microvesicles commonly lead to co-isolation and impurity of harvested Exo [14, 15]. In conventional approaches, such as ultracentrifugation, changes in morphology and functionality should not be neglected. Isolation of Exo via the high-speed pelleting method can lead to mechanical damage, exosomal membrane distortion, protein aggregation, lipoprotein contamination and low-rate purity [16]. Low-yield rate and alteration of exosomal cargo are possible in Exo collected via ultracentrifugation [17]. The isolation of Exo via ultracentrifugation can alter the final concentration of specific markers compared to the parent cells. Based on the previously conducted experiments, ultracentrifugal isolation of Exo led to a reduction of calnexin while the levels of CD81 and CD9 remained unchanged [18].

**Table 1:** List of MSC Exo clinical trials recorded up to September 2021 (available on https://clinicaltrials.gov/ct2/home)

Status	Study	Conditions	Source	Interventions	Phase
Not yet recruiting	Nebulization of MSC Exo in patients with acute respiratory distress syndrome	Acute respiratory distress syndrome	Allogenic MSCs	Low/medium/and high doses of MSC Exo	I and II
Completed	Aerosol inhalation of MSC Exo in healthy volunteers	Healthy	Allogenic adipose MSCs	1X to 8X concentration of Exo	I
Recruiting	Effect of umbilical MSCs Exo on dry eye in patients with cGVHD	Dry eye	Umbilical MSCs	10ug exosomal protein/drop	I and II
Completed	Inhalation of MSC Exo in severe Coronavirus pneumonia	Coronavirus	Allogenic adipose MSCs	2.0 × 10^8^ nanovesicles/3 ml	I
Recruiting	Allogenic MSC Exo in patients with acute ischemic stroke	Cerebrovascular disorders	Allogenic MSCs	Exo enriched by miR-124	I and II
Not yet recruiting	MSC Exo for multiple organ dysfunction syndromes after surgical repair of acute type A aortic dissection	Multiple organ failure	Umbilical MSCs	Intravenous administration of Exo (150 mg)	Not applicable
Recruiting	MSC Exo nebulization for the treatment of pulmonary infection	Drug-resistant	Allogenic adipose MSCs	8.0 × 10^8^ nanovesicles/3 ml	I and II
Active, not recruiting	MSC Exo for macular holes	Macular holes	Umbilical MSCs	Intraviterous injection of 20–50 μg/10 μl PBS	I
Not yet recruiting	MSC Exo for the treatment of acute respiratory distress syndrome (COVID-19)	COVID-19	Perinatal MSC Exo	Intravenous administration of MSC Exo	I and II
Not yet recruiting	MSC Exo on the therapy for intensively Ill children	Sepsis and critical illness	MSC Exo	ND	ND
Recruiting	MSC Exo in patients with Alzheimer's disease	Alzheimer Disease	Allogenic adipose MSCs	The nasal drip of low/medium and high doses of MSC Exo	I and II
Unknown	MSC Exo for induction of beta-cell mass in type I diabetes mellitus	Diabetes mellitus type 1	Cord blood MSC Exo	Intravenous injection	II and III
Enrolling by invitation	MSC Exo inhalation in COVID-19 associated pneumonia	SARS-CoV-2 pneumonia	MSC Exo	Inhalation of 0.5–2 × 10^10^ nanoparticles	II
Completed	MSC Exo inhalation in COVID-19 associated pneumonia	SARS-CoV-2 pneumonia	MSC Exo	Inhalation of 0.5–2 × 10^10^ nanoparticles	I and II
Not yet recruiting	MSC Exo for dystrophic epidermolysis bullosa	Dystrophic epidermolysis bullosa	Allogeneic bone marrow MSC Exo	Topical administration	I and II
Completed	MSC EVs inhalation in COVID-19 associated pneumonia	Covid19	Bone marrow MSC Exo	Intravenous	II
Not yet recruiting	MSC CM on enhancers of bone formation in bone grafting	Bone loss, osteoclastic	Autologous adipose MSC CM	Injection to the maxillary sinuses	I

Exo, exosomes; CM, condition media; MSC, mesenchymal stem cells; ND, none determined; EVs, extracellular vesicles

**Table 2:** Currently available methods for the isolation and purification of Exo

Method	Advantage	Disadvantage
Sequential ultracentrifugation	Low cost/contamination rate, approximate for large-volume samples	Time-consuming, Damage to exosomal integrity, genomic and proteomic aggregation, expensive equipment, the existence of other extracellular vesicles
Gradient ultracentrifugation	High-rate Exo purity, fractioning of extracellular vesicles into different subsets, large-volume samples	Time-consuming, mechanical damage to exosomal integrity, expensive equipment
Size exclusion chromatography	High-rate purification of Exo, suitable for Exo isolation without any damages, Fast and precise	Expensive equipment, the multi-step isolation procedure
Ultrafiltration	Fast and precise, relatively low cost	Damage to Exo, loss of Exo in samples, low to moderate purity
Polymer precipitation	High-rate purity, applicable for both small- and large-sized samples	Polymer contamination, protein aggregation, multi-step preparation, time-consuming
Immunoaffinity	Applicable for isolation of distinct Exo subpopulation, High-rate purity, Easy to use	Expensive, applicable for low-sized samples, the possibility of Exo damage, low-content Exo yield
Microfluidics	High-rate Exo purity, cost-effective	Applicable for low-sized samples

Storage is another critical issue related to Exo application in regenerative medicine. It is suggested that the lack of storage protocols can affect their size and composition. The intensity of these changes is higher at temperatures 4 and − 20 °C when compared to lower temperatures such as − 80 °C [19]. For instance, levels of CD63 and HSP70 are reduced when Exo are stored at higher temperatures such as 4 °C for 10 days [20]. Of note, the loss of exosomal cargo was higher at room temperature [20]. By increasing the temperature of storage conditions, the Exo population exhibits a more dispersed pattern. Many protocols used phosphate-buffered saline as a storage buffer for Exo for cryopreservation. The addition of some components such as trehalose into phosphate-buffered saline can prohibit Exo swelling [21]. Exosomal aggregation or cryodamage is another issue regarding the maintenance at below temperatures, leading to loss of Exo functionality after administration. The increase of freezing/thawing cycles can contribute to Exo aggregation and subcellular localization after the incubation with target cells [22]. The influence of storage pH has also been identified on Exo uptake by the cells. Exo maintained at pH values of 4 and 10 had better uptake levels rather than that of pH 7 [22]. Further elucidation of underlying mechanisms that lead to appropriate cryopreservation without affecting exosomal integrity and function is highly recommended.

The type of guidelines, methods and supplements used for parent MSC cultivation and donor-specific factors affect Exo profile and batch-to-batch variability [23]. It has been confirmed that the exosomal cargo is directly altered when the host cells are exposed to stressful conditions, leading to the increase of distinct factors and proinflammatory cytokines inside Exo [23]. Molecular identification of isolated Exo has indicated a close correlation between cargo type and levels with passage number. With an increasing passage number, membrane distribution of exosomal markers such as CD63 is reduced [24]. Cellular aging after long-term cultures and numerous passages can alter Exo production capacity and functionality [25]. Considering an initial seeding density of producing cells has an important impact on the quality and quantity of Exo. For example, seeding at higher densities can lead to insufficient Exo production and contamination with culture medium proteins while the cargo profile is also altered [24]. Commensurate with these descriptions, it is highly recommended to develop standard protocols to ensure the quality of isolated Exo from parent cells cultured on in vitro conditions. Along with the quality of parent cell culture and time of incubation, the medium composition is also responsible for Exo consistency. High-glucose medium supports large-sized Exo production with different protein content versus low glucose medium [26]. Other components used commonly in the culture medium are antibiotics and FBS. Prolonged culture of parent cells in the presence of specific antibiotics such as ciprofloxacin, a mycoplasma inhibitor, can increase a load of DNA on the Exo surface. This feature increases the possibility of Exo attachment to ECM protein such as fibronectin [25]. FBS is the source of exogenous Exo with potential bioactivities that can alter the physiology of culturing cells. To be honest, it is not clear to what extent current protocols are useful in the elimination of Exo from FBS, and therefore, the contamination of parent cell Exo and exogenous Exo should not be neglected. Noteworthy, the culture of cells in FBS-free conditions can induce extensive starvation and alter exosomal content [25].

**Fig. 2:**
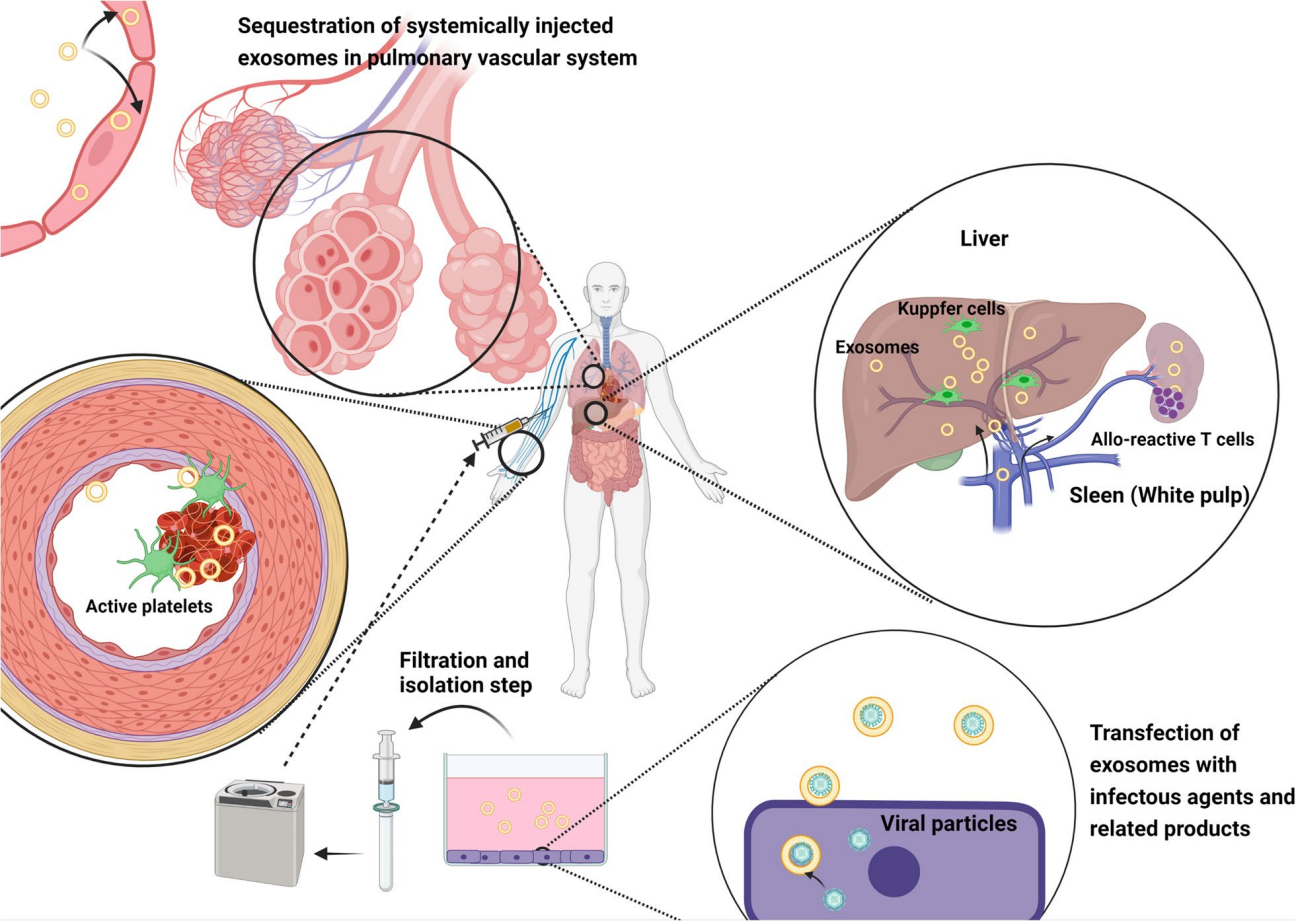
The injection of Exo may lead to allo-/xeno-reactive T cell responses via the activity of APCs located inside the hepatic and splenic tissues. In addition, systemically injected Exo can be sequestrated in pulmonary and hepatic vascular beds. Another issue regarding Exo application is the promotion of thrombosis in the vascular niche. Harboring infectious agents via Exo can lead to transmission of these particles into the in vivo milieu

Sterility is another critical issue in the context of Exo therapy (Fig. 2). Considering the approximately the same size among the different viruses and Exo, it is possible to mention that virions, viral products, toxins and bacteria-associated vesicles can be enriched in the Exo fraction. However, the presence of microbe and fungi in the Exo fraction is low due to their identical size and elimination following the filtration step (Fig. 2) [27]. It has been shown that Exo biogenesis machinery can be hijacked by specific retroviruses such as HIV-1 and HTLV-1 and these viruses use Exo as natural biocarriers to spread inside the body and circumvent the immune system responses [28]. The existence of virus-related genetics and proteins not only increases the possibility of infection but also alters the bioactivity of parent cells [28]. As a correlate, parent cells should be carefully monitored for dormant viral infections before application to the in vitro systems for large-scale production of Exo.

The possibility of thrombosis and hemostatic perturbations is the major concerns limiting the extensive application of Exo for therapeutic approaches via systemic route. It seems that the possibility of thrombosis is proportional to Exo concentration. The existence of phosphatidylserine and tissue factor closely correlates with the risk of thrombosis. Notably, large-sized Exo harbor higher pro-thrombotic factors compared to small-sized counterparts [29]. Therefore, one can hypothesize that Exo isolated from biofluids have higher thrombosis risk because of tissue factor and other procoagulant factors when compared to Exo are purified from parent cells in in vitro conditions. One reason would be that in systemic circulation Exo are heterogeneous and originated from several cell types mainly platelets and bone marrow megakaryocytes [30, 31]. It was suggested that these cells release Exo which are CD41 and high-mobility group box 1, promoting the possibility of vascular injury and thrombosis (Fig. 2) [32]. Therefore, it is mandatory to select appropriate sources for Exo purification according to therapeutic purposes. Contrary to common belief, allogenic Exo can be uptaken by APCs, leading to allo-reactive T cell responses [33]. Whether the intensity and duration of allo-reactive T cell response are more compared to whole-cell transplantation needs further investigation. Regarding trivial levels of recognition elements such as MHC-1 on the Exo surface and rapid cell entry event, it is logical to hypothesize that the exposure time of T lymphocytes and APCs with allo-reactive Exo is too short compared to transplant allogeneic MSCs (Fig. 2) [34, 35]. Although the existence of immune modulatory cytokines such as transforming growth factor-beta and interleukin-10 has been previously indicated in MSC-derived Exo, it should not be overlooked that repeated dosing of allogenic and especially xenogeneic Exo increases the likelihood of allo-/xeno-reactive responses [10]. Systemically administrated Exo can be eliminated via the activity of hepatic and splenic macrophages and pulmonary endothelial cells [35]. This phenomenon would result in the activation of APCs and failure to reach the proper dose of Exo into the target sites. Deciphering the underlying molecular pathways that lead to T cell and APC activation post-Exo administration is the subject of area.

## Conclusions

Progress in our knowledge about Exo has led to the understanding of their therapeutic properties and making them superior over their counterpart MSCs. Apart from differentiation capacity, MSCs exhibit noteworthy activity to secret soluble factors via Exo, accelerating the regeneration procedure possibly more than when committing to the functional mature cell types [36]. Understanding several aspects related to Exo properties have paved a way for efficient therapeutic strategies. Regarding their physicochemical properties, Exo have opened novel hopeful avenues for the alleviation of several pathologies. It is suggested that Exo can efficiently alter target singling molecules in the recipient cells, making them an appropriate therapeutic modulator in regenerative medicine.

Of note, the possibility of undesirable side effects is less, if not completely, compared to whole-cell transplantation (Table 3). Unique stability and size of Exo increase in vivo biodistribution rate with a low probability of aggregation after systemic injection. Moreover, the efficiency of delivery into the recipient cells can be selectively increased using surface and content modification strategies [37]. Despite these advantages, the lack of GMP-grade preparation protocols and definitions are major hurdles in the field of Exo therapy. It seems that critical technological considerations and definition systems are mandatory to obtain a better safety profile after Exo administration inside the in vivo conditions.

**Table 3:** Possible challenges related to Exo application in regenerative medicine

Challenges	Description
Isolation and purification methods	Lack of standard protocol for Exo isolation, leading to heterogeneity in Exo quality and quantity Impurity and contamination with non-Exo components The possibility of Exo damage
Biosafety	Contamination of Exo samples with infectious agents during isolation from biofluids or in vitro systems
Storage and maintenance	Temperature- and pH-dependent changes in Exo profile and physicochemical properties
In vivo administration	Possibility of thrombosis and biodistribution to nontarget organs Allo-reactive responses and elimination of Exo by the reticuloendothelial system
In vitro culture system condition	Effect of culture medium components on Exo production and cargo profile Contamination of Exo samples with exogenous Exo, protein and other biomolecules

## Data Availability

Not applicable.

## Abbreviations

APCs: Antigen-presenting cells
ECM: Extracellular matrix
Exo: Exosomes
FBS: Fetal bovine serum
GMP: Good manufacturing practices
HSP70: Heat shock protein 70
HIV-1: Human immunodeficiency virus-type 1
HTLV-1: Human T-cell leukemia virus-1
MHC-I: Major histocompatibility complex I
MSCs: Mesenchymal stem cells
